# A Simple Pre-Exposure Prophylaxis (PrEP) Optimization Intervention for Health Care Providers Prescribing PrEP: Pilot Study

**DOI:** 10.2196/formative.8623

**Published:** 2018-01-16

**Authors:** Parya Saberi, Beth Berrean, Sean Thomas, Monica Gandhi, Hyman Scott

**Affiliations:** ^1^ Center for AIDS Prevention Studies University of California, San Francisco San Francisco, CA United States; ^2^ Department of Medicine University of California, San Francisco San Francisco, CA United States; ^3^ Infectious Diseases and Global Medicine Division Department of Medicine University of California, San Francisco San Francisco, CA United States; ^4^ Bridge HIV San Francisco Department of Public Health San Francisco, CA United States

**Keywords:** pre-exposure prophylaxis, PrEP, health care providers, HIV, technology, panel management

## Abstract

**Background:**

Pre-exposure prophylaxis (PrEP) has been shown to be highly effective for the prevention of HIV in clinical trials and demonstration projects, but PrEP uptake and adherence outside of these settings in the United States has been limited. Lack of knowledge and willingness of health care providers (HCPs) to prescribe PrEP is an important barrier to implementation.

**Objective:**

The objective of this study was to describe and examine the feasibility and acceptability of a PrEP Optimization Intervention (PrEP-OI) targeted at HCPs. The ultimate purpose of this intervention was to increase PrEP uptake, adherence, and persistence among those at risk for HIV acquisition.

**Methods:**

This intervention included the following: (1) a Web-based panel management tool called PrEP-Rx, which provides comprehensive HIV risk assessment, automates reminders for follow-up, and reports patients’ history of PrEP use; and (2) centralized PrEP coordination by a clinical support staff member (ie, the PrEP coordinator) who can identify individuals at risk for HIV, provide medical insurance navigation, and support multiple HCPs. Feasibility was evaluated based on HCPs’ ability to log in to PrEP-Rx and use it as needed. Acceptability was assessed via individual formative qualitative interviews with HCPs after 1 month of the intervention.

**Results:**

The intervention was feasible and acceptable among HCPs (N=6). HCPs identified system-level barriers to PrEP provision, many of which can be addressed by this intervention. HCPs noted that the intervention improved their PrEP knowledge; increased ease of PrEP prescription; and was likely to improve patient engagement and retention in care, enhance communication with patients, and improve patient monitoring and follow-up.

**Conclusions:**

Given the critical role HCPs serve in disseminating PrEP, we created an easy-to-use PrEP optimization intervention deemed feasible and acceptable to providers. Further research on this tool and its ability to impact the PrEP continuum of care is needed.

## Introduction

Pre-exposure prophylaxis (PrEP) has been shown to be highly effective for the prevention of HIV in randomized clinical trials [1-6] and demonstration projects [7-9], but PrEP uptake in the United States has not been concomitant with need [10-12]. Despite data indicating nearly 80,000 individuals starting PrEP by the end of 2015 [13], the US Centers for Disease Control and Prevention (CDC) estimates that there are over 1.2 million adults in the United States at substantial risk for HIV acquisition [14]. Identified barriers to PrEP implementation include the individual’s awareness of and willingness to take PrEP and their access to health care and the knowledge and willingness of health care providers (HCPs) to prescribe PrEP [10,15-20]. The prevention of new HIV infections remains a critical public health priority, and PrEP is an essential [21], yet underused, component of the HIV prevention toolkit. As HCPs are important gatekeepers for biomedical HIV prevention efforts in clinical settings and provider knowledge and self-efficacy are important predictors of offering testing [22,23] and prevention modalities, HCPs require support and guidance to optimize the clinical and public health impact of PrEP.

Numerous surveys have been conducted to evaluate HCPs’ knowledge of PrEP, barriers to prescribing PrEP, and real-world challenges [10,12,19,20,24-28]. In one survey of Emerging Infections Network members in the United States and Canada [12], only 9% had provided PrEP, and despite the availability of comprehensive guidelines from the CDC [29-32], PrEP practices were variable. When physicians who had indicated that they would not provide PrEP were asked about their barriers to prescribing this medication, 77% stated that they worried about adherence and the risk for future resistance, 57% were concerned about cost and reimbursement issues, 53% did not want to use potentially toxic drugs in healthy persons, and 53% felt there was insufficient evidence for the efficacy of real-world PrEP. A recent paper examined family planning providers’ knowledge of and attitudes toward PrEP [17]. Despite the CDC’s definition of HIV prevention as a core family planning service, the authors noted that only 38% correctly defined PrEP, 37% understood the effectiveness of PrEP, and only 36% of respondents consulted PrEP guidelines. Most surveys have concluded that more education, provision of guidelines for real-world PrEP delivery, and interventions are needed to provide accurate data and optimize PrEP implementation [10,12,19,20,24,25,27] nationwide. In a recent national survey from 2009 to 2015, only 66% of primary care clinicians were aware of PrEP, although, once defined, 91% indicated a willingness to prescribe PrEP for patients at risk for HIV and expressed an interest in being educated further about PrEP [33].

A descriptive report on the early experiences with PrEP uptake and delivery in San Francisco identified the following priority steps for HCPs to address PrEP delivery barriers and to maximize public health impact: (1) increase PrEP knowledge among HCPs and (2) expand PrEP access by training HCPs and developing tools to facilitate PrEP delivery in clinical practices [34]. Moreover, based on the framework of the PrEP care continuum [35], increasing PrEP uptake will require HCP education, tools to assess sexual risk, and systems to minimize provider burden. On the basis of these suggestions, innovative and effective approaches are needed to support PrEP implementation by HCPs regardless of their level of experience.

Therefore, we sought to develop a PrEP Optimization Intervention (PrEP-OI) targeted at HCPs with the goal of ultimately improving the PrEP care continuum. PrEP-OI includes the following: (1) an integrated Web-based panel management tool, called PrEP-Rx, which provides structured HIV risk assessment for individuals of all genders and HIV risk factors, automates reminders for laboratory testing and follow-up appointments, and reports patients’ current and history of PrEP use; and (2) centralized PrEP coordination overseen by a clinical support staff member (referred to as the PrEP coordinator) who can support multiple HCPs and identify individuals at high risk for HIV through various methods, including structured behavioral surveys, direct patient contact, or by reviewing registries for sexually transmitted infections. Here we describe PrEP-OI and the results of a pilot study to examine its feasibility and acceptability among HCPs prescribing PrEP.

## Methods

We developed PrEP-OI (PrEP-Rx and PrEP coordinator) with direct input from HCPs and conducted a pilot study over 1 month to examine intervention feasibility and acceptability in a San Francisco clinic offering PrEP. Our interdisciplinary team consisted of HIV researchers from the University of California, San Francisco (UCSF), Center for AIDS Prevention Studies (CAPS), HIV clinicians from San Francisco General Hospital’s HIV clinic (Ward 86), and technology design/development experts (the Information Services Unit) from UCSF. We received funding from the UCSF Center for AIDS Research (grant number P30 AI027763) to design and develop PrEP-Rx and received approval from the UCSF Institutional Review Board to conduct our pilot study.

### Description of the Pre-Exposure Prophylaxis Optimization Intervention

**Figure 1:**
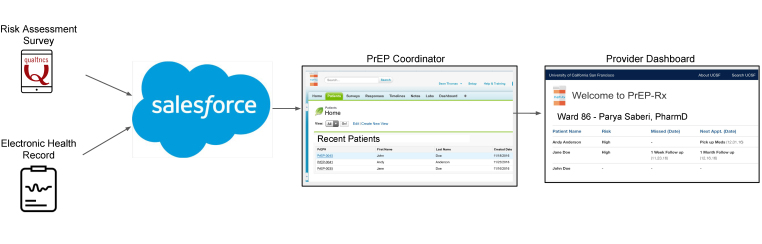
Architecture of PrEP-Rx.

**Figure 2:**
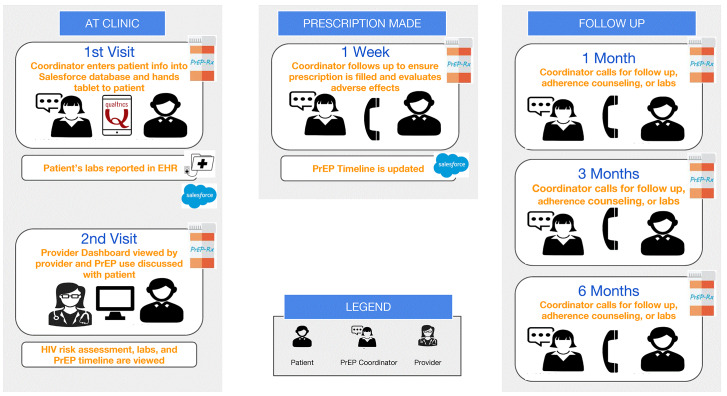
Hypothetical Clinic Work Flow.

PrEP-Rx was created using a Salesforce backend, with the potential to integrate with electronic health record (EHR) for laboratory values and demographic data, and a Qualtrics survey was used to assess risk among potential PrEP users (Figure 1). The hypothetical clinic workflow (Figure 2) demonstrated the need for 3 main components: (1) a mechanism to comprehensively assess HIV risk for individuals of all genders and HIV risk factors; (2) automated reminders for upcoming and overdue laboratory monitoring and follow-up visits to assess adherence and adverse effects and to perform risk reduction counseling; and (3) a website with PrEP educational material (eg, guidelines, publications, conference proceedings) for the ongoing education and training of HCPs.

A risk assessment questionnaire was created by Drs Saberi and Scott using available published data to capture an individual’s risk profile. The CDC risk index [36] was used for men who have sex with men (MSM). With input from behavioral risk assessment experts at the UCSF Transgender Centers of Excellence and CAPS, we modified this assessment to include questions specific for transgender men and women. Next, using data from the CDC guidelines, we added risk assessment questions for heterosexual men and women [29], and with information from a publication by Smith et al [37], we added questions regarding injection drug use (IDU) risk. In the absence of a uniform rating scale for MSM, IDU, and heterosexual sex, we modified this tool to include 3 tiers of risk: (1) low, (2) medium, and (3) high (Table 1). The CAPS Community Advisory Board reviewed all questions for clarity and cultural appropriateness. We programed this questionnaire into Qualtrics and integrated the responses with the Salesforce database (Figure 1).

**Figure 3:**
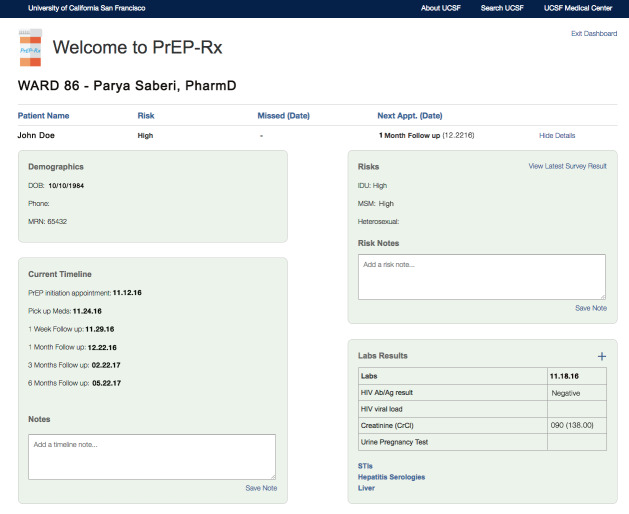
Provider Dashboard.

The provider dashboard is a method of summarizing patients’ pertinent information (ie, HIV risk category, prior PrEP use, and laboratory data) in one simple format for the HCP (Figure 3). This dashboard consists of 4 components (in separate boxes) for each patient: (1) demographics (including patient’s name, medical record number, date of birth, and telephone number); (2) PrEP timeline(s) (summary of current and prior PrEP periods of use, including dates of initial PrEP appointment, follow-up appointments, laboratory visits, and reasons for prior PrEP discontinuations, if applicable); (3) risk categories (summary of risk based on the risk assessment questionnaire and the risk assessment category); and (4) laboratory data required for PrEP initiation (including the last 2 laboratory results on file). The HCP can type a note in the *PrEP Timeline* and *Risk* sections to reference in future follow-up visits.

**Table 1:** Three tiers of HIV risk in PrEP Rx.

Risk	Men who have sex with men	Injection drug use	Heterosexual sex
High risk	Age: 18-28 years 10 male sex partners in the past 6 months Any receptive anal sex with a man without a condom >1 HIV+ or HIV status unknown male partner in the past 6 months Any commercial sex work in the past 6 months Any sexually transmitted infection If 2 or more medium-risk factors	Age: <30 years If NOT in methadone maintenance program in the last 6 months If composite injection score (inject heroin, inject cocaine, share cooker, share needles, visit shooting gallery) >1 If 2 or more medium-risk factors	10 opposite-sex sex partners in the past 6 months (For men only) Any sex without a condom with a woman at high risk for HIV (eg, IDU^a^) or HIV+ or HIV status unknown (For women only) Any sex without a condom with a partner at high risk for HIV (eg, IDU or bisexual male) or HIV+ or HIV status unknown Any commercial sex work in the past 6 months If 2 or more medium-risk factors
Medium risk	Age: 29-40 years If composite injection score (inject heroin, inject cocaine, share cooker, share needles, visit shooting gallery) =1 1 HIV+ or HIV status unknown male partner in the past 6 months Use of methamphetamine in the past 6 months >1 insertive anal sex without a condom with a man who was HIV+ or HIV status unknown unknown Heavy alcohol use (5-7 days a week and drinks per day ≥4 or 1-7 days per week and ≥6 drinks per day) Use of cocaine/crack or poppers	Age: 30-39 years 6-10 male partners in the past 6 months	6-10 opposite-sex sex partners in the past 6 months Any sexually transmitted infection
Low risk	Everyone else	Everyone else	Everyone else

^a^IDU: injection drug use.

The PrEP coordinator is responsible for supporting both HCPs and patients in this intervention. The PrEP coordinator enters the patient’s medical record number into PrEP-Rx, which pulls demographic information and laboratory data from the clinic’s EHR (Figure 1). During the patient’s visit with the PrEP coordinator, he/she is handed a tablet to respond to the risk assessment questionnaire. On the basis of the patient’s responses, HIV risk categories are generated and displayed on the provider dashboard, along with the patient’s demographics, laboratory data, and current and/or prior PrEP history (ie, PrEP timeline). The patient then attends an appointment with the HCP where they can discuss the patient’s risk and laboratory test results, desire to initiate PrEP (which can be recorded in the notes section of the provider dashboard), and any questions or concerns about PrEP. On the basis of the PrEP prescription date, PrEP-Rx auto-generates a list of dates for follow-up laboratory tests and appointments based on CDC guidelines. As a result, the PrEP coordinator receives notifications via emails about upcoming or past due visits, allowing her/him to notify the patient and mark these tasks as “done” upon completion.

Finally, to create a system for the ongoing education of HCPs, we created the PrEP-Rx website to host the PrEP knowledge base. The purpose of the knowledge base is to provide access to the most recent publications, ongoing PrEP research, and conference proceedings. All components of PrEP-Rx are built in an environment that is compliant with the Health Insurance Portability and Accountability Act and meet the highest level of security requirements.

### Pilot Study

We conducted a 1-month pilot study to evaluate the feasibility, acceptability, and usability of PrEP-OI at a publicly funded safety-net clinic in San Francisco offering PrEP. Feasibility was evaluated based on HCPs’ ability to log in to PrEP-Rx and use it as needed (based on Google Analytics). The acceptability of PrEP-Rx was assessed in one-on-one formative qualitative interviews with providers who had used PrEP-Rx for 1 month along with PrEP coordination. The main goal of these formative interviews was for providers to give feedback and to assist us in optimizing PrEP-Rx through our iterative design. Questions included the following: (1) experiences with PrEP-Rx (ease/difficulty with initial login and navigation, glitches, and opinions on its interface), (2) how it may have impacted the HCP’s knowledge and ability to prescribe PrEP, (3) experience with the PrEP coordinator, (4) what aspects of PrEP-OI HCPs found favorable and unfavorable, (5) how this intervention can be further streamlined to fit within their workflow, (6) whether they would recommend this intervention to their peers, and (7) whether they would consider continuing to use it in their clinical practice. Usability was evaluated based on a modified System Usability Scale [38], which evaluates the HCP’s desire to use PrEP-Rx, complexity/ease of use, need for technical support, confidence with use, and speed of learning to use PrEP-Rx. Items are scored on a scale of 1-5 (strongly disagree to strongly agree) and summed to provide an overall score ranging from 0 to 100.

Formative qualitative interviews were audio-recorded, transcribed verbatim, and summarized by the first author. Broad themes were then identified and entered into a matrix using Microsoft Excel where columns and rows represented themes and participants, respectively, to facilitate data analysis and the identification of patterns in the distribution of themes [39]. The first author categorized each interview using this matrix, and themes were discussed with the second, third, and last authors. On the basis of these discussions, modifications to the design of PrEP-Rx were identified.

## Results

A total of 6 HCPs gave us feedback on their experience with PrEP-OI after 1 month of use in December 2016. HCPs were physicians 83% (5/6) and nurse practitioners 17% (1/6), female 67% (4/6), white 50% (3/6), working in an HIV clinic 100% 6/6), with a mean of 13 years (range 4-17 years) of experience providing care for individuals living with HIV. Approximately 67% (4/6) and 33% (2/6) provided HIV care for 20-49 and greater than or equal to 50 patients living with HIV, respectively. Of the HCPs, 2 (33%) did not have an active PrEP patient at the time of the interview, 3 (50%) were providing PrEP to 1-9 patients, and 1 (17%) was providing PrEP to 10-19 patients.

Using the System Usability Scale, HCPs gave PrEP-Rx a score of 94.6 (standard deviation 6.4). This meant that they strongly agreed that they (1) would like to use PrEP-Rx, (2) thought it was easy to use and did not need to learn a lot before getting started, (3) found the functions well integrated and consistent with the goals of the project, and (4) felt confident using PrEP-Rx without technical support.

The initial meeting with each HCP to describe the purpose of PrEP-Rx, to ensure ability to log in, and to answer any questions took approximately 20 min. All HCPs stated that their initial log in was easy and fast and that the use of a single sign-on service made it easy for them to log in without needing to remember an additional username and password. Of the providers, 1 had difficulty with initial log in due to the use of an old Web browser version. On the basis of Google Analytics, during the 1 month of use, users had 74 PrEP-Rx sessions, and the average session duration was 6:08 minutes. None of the users contacted us during the 1 month of the pilot about inability to log in, glitches, or problems accessing their dashboard.

In one-on-one formative qualitative interviews, HCPs discussed barriers that they had experienced in providing PrEP and gave constructive feedback on improvements to PrEP-Rx. Barriers were categorized into patient-, system-, and societal-level and summarized in Table 2. HCPs identified the biggest challenge as missed medical visits by patients on PrEP.

HCPs stated that the PrEP-Rx tool is easy to use (HCPs #1, #2, and #3), “clean” (HCPs #1 and #3), “intuitive” (HCP #3), “soothing” (HCP #6), and organized using “boxes so you know where to look for stuff” (HCP #1).

...clean, clear, organized in a manner which I expect to see things, not a lot of wasted space...

HCP #3

They liked the simple look of PrEP-Rx, stating “I dislike websites that are too slick” (HCP #2).

HCPs unanimously reported that PrEP-Rx has the potential to improve their level of information regarding PrEP, capacity for PrEP provision, and/or motivation to prescribe PrEP. Table 3 summarizes HCPs’ statements of the areas that PrEP-OI could impact.

HCPs requested the following modifications to PrEP-Rx: (1) in the Demographics section, the addition of patient’s email address and a demographics notes section; (2) in the PrEP Timeline section, addition of the date that the PrEP prescription was written (if different from the initial PrEP appointment); (3) in the Laboratory section, the permanent display of the baseline laboratory values (ie, before PrEP initiation), information about laboratory normal ranges or cutoffs for PrEP prescription, and a laboratory notes section; (4) in the Risk Assessment section, addition of guidance around PrEP medication dosing frequency and number of refills to prescribe upon PrEP initiation and availability of the risk assessment questionnaire in Spanish; (5) on the PrEP-Rx website, addition of pivotal PrEP studies and educational handouts or information that the HCP can give to patients to answer questions (eg, what is PrEP and why is it being prescribed, how is PrEP taken, what to do in case of intolerance, and who to call in case of emergencies); and (6) addition of a new section to track PrEP adherence and persistence.

None of the HCPs had any major concerns regarding the privacy and security of the data; all wanted to continue using PrEP-Rx, and would recommend it to other HCPs and for use in other countries.

I would love to have more opportunity to use it...things like this will help us overcome a lot of limitations in our current system, so I'd love to continue using it.

HCP #6

I've been thinking in terms of PrEP roll out in places where Internet access is challenging...so there’s so much happening in terms of more countries approving PrEP, like Kenya and South Africa. If you think of, in terms of clinics that are already so over-burdened, and what can be helpful for providers, even if it’s a lay health worker who is asking questions on a tablet so by the time they get to the provider...I can see it being translatable on a tablet in other countries.

HCP #6

**Table 2:** Barriers to the provision of pre-exposure prophylaxis as identified by health care providers.

Category and subcategory		Example quotes
**Patient-specific**		
	Lack of PrEP^a^ information or misinformation, especially in monolingual Spanish-speaking patients or immigrants	*Uptake in mono-lingual Spanish-speaking patients is very low. It’s a hard concept to grasp in terms of: “if I’m not sick, why should I take a pill?”* [HCP^b^#1]
	Patients less concerned about risk of HIV acquisition due to being in a long-term partnership without HIV transmission from seropositive partners	...*for other partners of patients of mine, who have chosen not to start, there is some feeling of perhaps being at lower risk because of long-term partnerships and no transmission so far either through suppressed viral load, condoms, or both...* [HCP #6]
	PrEP nonadherence, nonpersistence, and loss-to-follow-up possibly due to mental health issues, substance use, food insecurity, or homelessness	*...potential PrEP clients are on the highly vulnerable end of the spectrum. Several of them who were scheduled to see me for PrEP evaluation never showed up and a number, who showed up for the first visit, never came back for follow-up.* [HCP #2]
		...*getting them in to their second visit, one-month visit and three-month visit is challenging. They are first eager but then follow-up is problematic. It may be due to substance use.* [HCP #4]
**System-specific**		
	Fractionated medical coverage, especially in those on Medi-Cal and with younger patients	*We have a lot of unknowns...We have a lot challenges from an operational public health stand point like how do we keep people accessing PrEP regardless of where they are. Every door should be right door, so people don't lose access to PrEP.* [HCP #3]
		*So, if someone has insurance, they have to renew it, particularly if they have Medi-Cal, I believe every 6 months. So, they often will receive a letter and if they don’t respond to that letter, they’re just dropped. So, they don’t really realize what has happened before it’s too late. Some pharmacists are great and they’ll figure out a bridge while we figure out the insurance, but in many cases people will go without PrEP for weeks to months while we figure out a way to get the insurance back up. So, there’s the hassle factor of reinitiating PrEP that for a lot of people is a big barrier.* [HCP #3]
	HCPs not addressing PrEP or HIV risk	*...if you're a providers in a primary care center or another clinic, do they address PrEP with their patients? and I think not in many cases...some of them don’t know about it, even though it's been available...it’s one more thing you need to deal with and when you have a lot of things on your plate and you only have 15 minutes in many cases, you’re going to pick the things you’re responsible for: the high blood pressure and diabetes...how many primary care providers actually ask their patients if they're sexually active, have a sexual partner...there’s a lot of assumptions made and, unfortunately, it's a topic that if the patient does not bring up, the provider thinks everything is okay...* [HCP #1]
	Ability to identify and engage people at high risk for HIV acquisition	*The first, I supposed, is getting people in for PrEP...identifying people at risk and actually getting them into clinic.* [HCP #4]
	Delay in getting laboratory results before PrEP initiation	*I will say that it’s very rare for someone to have tested before they come even though they’ve usually been given lab orders...but they rarely come in before their first meeting with me...so there is often a delay, so I’ll say: “...we’ll call you in a day or two...and we’ll send in the prescription if everything is looking good.” So...there’s a delay and we have to go chasing them...Usually it’s okay but it would be much more satisfying if we could give them the prescription knowing their results...ideally, they’d do labs in advance and be able to start if everything looks appropriate...* [HCP #4]
**Societal-specific**		
	Stigma associated with being on medications for HIV	...*this woman who couldn’t get PrEP, some of it was because of stigma and also concern for, “Is this medicine for anything other than HIV if I go to the pharmacy and pick it up?”* [HCP #6]

^a^PrEP: pre-exposure prophylaxis.

^b^HCPs: health care providers.

**Table 3:** Potential impacts of the pre-exposure prophylaxis (PrEP) Optimization Intervention on the provision of PrEP.

Intervention component and impact on PrEP^a^ prescribing		Example quotes
**PrEP-Rx**		
	Knowledge base providing quick references and information related to PrEP	...*it streamlines all the important elements that you’d want to know for a discussion with the patient. It lays out things clearly so it’s easy to see all the things in follow-up. Timeline gives a summary and walks you through the steps and is laid out in a structured way.* [HCP #3]
	Ability to provide all necessary data for all patients on PrEP in 1 comprehensive streamlined format, resulting in increased ease of PrEP prescription and being more effective	*...being able to look at the information on risk and timeline and having patients teed up, is very helpful and so much easier than current HER...it makes me so much more effective as a provider of PrEP.* [HCP #4]
	Allowing for the PrEP coordinator to function more effectively	*The survey goes into so much detail that would take a really long time as a provider to go into and it would be unlikely to get into this exact level of detail without a fairly involved visit especially for someone you’re meeting for the first time...We’re always focused on time as providers, so overcoming that hurdle of how many different screens do I need to click through to figure out...all their labs, trying to have all their follow-up, and really just the survey being so detailed. That would overcome a lot of the energy to do this in a reasonable amount of time for the provider and the patient.* [HCP #6]
**PrEP coordinator**		
	Increasing patient engagement in health care and retention	*[the PrEP coordinator] is the “glue” as far as getting the person in and encouraging them to come in...it is undoable without [the PrEP coordinator].* [HCP #4]
	Improving communication between health care providers and patients	...*having multiple touch points for the patient can be really helpful...the more likely you are to get someone engaged and sustained...* [HCP #6]
**PrEP Rx + PrEP coordinator**		
	Helping keep patients healthy by improving monitoring and follow-up	*...it motivates in as much as it’s linked to the patient. Because for providers that’s where our motivation is: to keep our patients healthy and happy...I think that thinking about how this is helping my patients and how this is helping reduce their HIV risk, is a motivation for me...* [HCP #3]
	Reducing loss to follow-up	*It makes me capable and motivates me because I know my patients are getting good care and know when my patients have fallen out of care.* [HCP #4]

^a^PrEP: pre-exposure prophylaxis.

## Discussion

We created the PrEP-OI, which includes a clinical staff member supporting HCPs (ie, the PrEP coordinator) and an easy-to-use Web-based tool (ie, PrEP-Rx), with guidance from HCPs. We then conducted a pilot study to examine the feasibility and acceptability of this intervention among 6 HCPs prescribing PrEP with varying levels of PrEP experience. The results of our pilot study reveal that this intervention is feasible to implement in clinical settings, and acceptable among HCPs regardless of their level of PrEP experience. Overall, HCPs identified several system-level barriers to PrEP provision (eg, HCPs not addressing HIV risk in routine practice, identification and engagement of people at high risk for HIV acquisition, and insurance navigation), many of which can be addressed with PrEP-OI. Additionally, the intervention was noted to improve HCPs’ PrEP knowledge and information and increase ease of PrEP prescription, and deemed likely to increase patient engagement and retention in care, improve communication between HCPs and patients, and improve quality of PrEP monitoring.

HCPs rated PrEP-Rx highly on the System Usability Scale and had favorable reviews of its look, feel, and functionality. There were no major privacy concerns, and all HCPs wanted to continue its use in their clinical practice. HCPs requested several additions to the Demographics, PrEP Timeline, Risk Assessment, and Laboratory sections, as well as a new section on PrEP adherence and additional information for patients and HCPs on the PrEP-Rx website.

Our intervention was pilot-tested in a clinic that employs panel management strategies. Panel management is defined as a “set of tools and processes for population care that are applied systematically at the level of a primary care panel” [40,41]. It is an approach to ensure that patients are up-to-date on their care and can receive additional support if required. Panel management can involve clinical support staff using chronic disease registries, EHRs, and other data-reporting tools to identify missed opportunities for disease prevention and treatment. These support staff are the liaison between the HCP and the patient and communicate and reinforce recommendations from HCPs or guidelines to the patient. Panel management strategies have been used in many settings to improve rates of vaccination, bone density screening for the elderly, hypertension treatment, and so on [42,43]. Successful panel management programs employ computerized clinical support tools to provide relevant care reminders, data registries, and performance feedback [44]. These electronic clinical tools are associated with improved health care management [45]. In our study, we leverage panel management strategies including a staff member (ie, PrEP coordinator) and a clinical support tool (ie, PrEP-Rx) to serve a goal of enhancing PrEP initiation and appropriate monitoring and follow-up. To our knowledge, this is the first study examining these strategies in the setting of HIV PrEP.

The development of PrEP-OI highlights the importance of joint efforts between academia, industry, and community partners. Using the framework of the PrEP care continuum [35], we chose to focus on the role of the HCP in being educated about PrEP, screening for high-risk behaviors, prescribing PrEP, and conducting ongoing monitoring and follow-up with the help of the PrEP coordinator. The PrEP coordinator is responsible for verifying patients’ insurance and eligibility status, scheduling appointments, evaluating HIV risk using the risk assessment questionnaire, following up with patients, and conducting medication and adherence counseling. PrEP-Rx is an electronic tool that provides education for HCPs, allows for the assessment of HIV risk in a comprehensive manner, tracks monitoring and follow-up visits, and summarizes details related to HIV risk, pertinent laboratory results, and patients’ current and/or prior PrEP use.

Limitations of our study include a small sample of HCPs at a clinic in a single geographic region over a short timeframe. Clinicians in this clinic were HIV specialists and received support from the PrEP coordinator. Therefore, our results may not be generalizable to other cities or clinics. A randomized trial of several primary care clinics in the San Francisco Bay Area to examine PrEP uptake and persistence over longer periods of time with this PrEP intervention is in progress.

PrEP-OI, including a PrEP coordinator and PrEP-Rx, has the potential to enhance PrEP uptake, adherence, and persistence along the PrEP care continuum [35] and improve HCPs’ knowledge and prescribing practices. Studies have highlighted the cost-effectiveness of PrEP in target populations [46-48]; however, given the high upfront costs, PrEP scale-up may not be feasible in many settings. Additionally, despite an increase in PrEP uptake in San Francisco, most recent data indicate that demand currently exceeds the supply of providers who prescribe PrEP [49]. Therefore, the automation of risk assessment, risk calculation, and reminders for monitoring and follow-up in addition to the assistance of a designated clinic staff to oversee these efforts and assist with establishment of medical coverage has the potential to reduce costs associated with clinician time and clinic resources.

Due to expanding access to Web-based and mobile technologies for decision support, HCPs are increasingly using online resources [50]. These technologies have been in the form of remote monitoring technologies [51], computerized decision support systems [52], and mHealth technologies (including geographic information systems) [53]. Many studies have reported providers’ acceptance of online and mobile technologies and demonstrated promising results, such as improved retention in care for their patients. Therefore, providing support to HCPs is essential for effective PrEP implementation as they are the supply link for those interested in initiating PrEP. PrEP-OI has the potential for improving the efficiency and quality of this “supply” link by assisting HCPs in prescribing PrEP.

As a next step, we will evaluate the efficacy of PrEP-OI to increase PrEP prescriptions and PrEP persistence through a stepped-wedge design among primary care clinics in San Francisco. Given the need for efficient and targeted identification of those at high risk for HIV acquisition, establishment of medical coverage, standardized and comprehensive HIV risk assessment, efficiency in HCP-patient communication around HIV risk and PrEP use, and appropriate monitoring and follow-up, the proposed PrEP-OI has the potential to significantly enhance the HIV PrEP continuum of care. If successful, our goal is to implement this intervention in other health systems, in settings outside of San Francisco, and in international settings.

## Abbreviations

CAPS: Center for AIDS Prevention Studies
CDC: US Centers for Disease Control and Prevention
EHR: electronic health record
HCP: health care provider
IDU: injection drug use
MSM: men who have sex with men
PrEP: pre-exposure prophylaxis
PrEP-OI: PrEP Optimization Intervention
UCSF: University of California, San Francisco

## References

[1] Grant RM, Lama JR, Anderson PL, McMahan V, Liu AY, Vargas L, Goicochea P, Casapía M, Guanira-Carranza JV, Ramirez-Cardich ME, Montoya-Herrera O, Fernández T, Veloso VG, Buchbinder SP, Chariyalertsak S, Schechter M, Bekker LG, Mayer KH, Kallás EG, Amico KR, Mulligan K, Bushman LR, Hance RJ, Ganoza C, Defechereux P, Postle B, Wang F, McConnell JJ, Zheng JH, Lee J, Rooney JF, Jaffe HS, Martinez AI, Burns DN, Glidden DV, iPrEx Study Team (2010). Preexposure chemoprophylaxis for HIV prevention in men who have sex with men. N Engl J Med.

[2] Thigpen MC, Kebaabetswe PM, Paxton LA, Smith DK, Rose CE, Segolodi TM, Henderson FL, Pathak SR, Soud FA, Chillag KL, Mutanhaurwa R, Chirwa LI, Kasonde M, Abebe D, Buliva E, Gvetadze RJ, Johnson S, Sukalac T, Thomas VT, Hart C, Johnson JA, Malotte CK, Hendrix CW, Brooks JT, TDF2 Study Group (2012). Antiretroviral preexposure prophylaxis for heterosexual HIV transmission in Botswana. N Engl J Med.

[3] Baeten JM, Donnell D, Ndase P, Mugo NR, Campbell JD, Wangisi J, Tappero JW, Bukusi EA, Cohen CR, Katabira E, Ronald A, Tumwesigye E, Were E, Fife KH, Kiarie J, Farquhar C, John-Stewart G, Kakia A, Odoyo J, Mucunguzi A, Nakku-Joloba E, Twesigye R, Ngure K, Apaka C, Tamooh H, Gabona F, Mujugira A, Panteleeff D, Thomas KK, Kidoguchi L, Krows M, Revall J, Morrison S, Haugen H, Emmanuel-Ogier M, Ondrejcek L, Coombs RW, Frenkel L, Hendrix C, Bumpus NN, Bangsberg D, Haberer JE, Stevens WS, Lingappa JR, Celum C, Partners PrEP Study Team (2012). Antiretroviral prophylaxis for HIV prevention in heterosexual men and women. N Engl J Med.

[4] Choopanya K, Martin M, Suntharasamai P, Sangkum U, Mock PA, Leethochawalit M, Chiamwongpaet S, Kitisin P, Natrujirote P, Kittimunkong S, Chuachoowong R, Gvetadze RJ, McNicholl JM, Paxton LA, Curlin ME, Hendrix CW, Vanichseni S, Bangkok TSG (2013). Antiretroviral prophylaxis for HIV infection in injecting drug users in Bangkok, Thailand (the Bangkok Tenofovir Study): a randomised, double-blind, placebo-controlled phase 3 trial. Lancet.

[5] Molina J, Capitant C, Spire B, Pialoux G, Cotte L, Charreau I, Tremblay C, Le GJ, Cua E, Pasquet A, Raffi F, Pintado C, Chidiac C, Chas J, Charbonneau P, Delaugerre C, Suzan-Monti M, Loze B, Fonsart J, Peytavin G, Cheret A, Timsit J, Girard G, Lorente N, Préau M, Rooney JF, Wainberg MA, Thompson D, Rozenbaum W, Doré V, Marchand L, Simon M, Etien N, Aboulker J, Meyer L, Delfraissy J (2015). On-demand preexposure prophylaxis in men at high risk for HIV-1 infection. N Engl J Med.

[6] McCormack S, Dunn DT, Desai M, Dolling DI, Gafos M, Gilson R, Sullivan AK, Clarke A, Reeves I, Schembri G, Mackie N, Bowman C, Lacey CJ, Apea V, Brady M, Fox J, Taylor S, Antonucci S, Khoo SH, Rooney J, Nardone A, Fisher M, McOwan A, Phillips AN, Johnson AM, Gazzard B, Gill ON (2016). Pre-exposure prophylaxis to prevent the acquisition of HIV-1 infection (PROUD): effectiveness results from the pilot phase of a pragmatic open-label randomised trial. The Lancet.

[7] Baeten JM, Heffron R, Kidoguchi L, Mugo NR, Katabira E, Bukusi EA, Asiimwe S, Haberer JE, Morton J, Ngure K, Bulya N, Odoyo J, Tindimwebwa E, Hendrix C, Marzinke MA, Ware NC, Wyatt MA, Morrison S, Haugen H, Mujugira A, Donnell D, Celum C, Partners Demonstration Project Team (2016). Integrated delivery of antiretroviral treatment and pre-exposure prophylaxis to HIV-1-serodiscordant couples: a prospective implementation study in Kenya and Uganda. PLoS Med.

[8] Hosek SG, Rudy B, Landovitz R, Kapogiannis B, Siberry G, Rutledge B, Liu N, Brothers J, Mulligan K, Zimet G, Lally M, Mayer KH, Anderson P, Kiser J, Rooney JF, Wilson CM, Adolescent Trials Network (ATN) for HIVAIDS Interventions (2017). An HIV preexposure prophylaxis demonstration project and safety study for young MSM. J Acquir Immune Defic Syndr.

[9] Liu AY, Cohen SE, Vittinghoff E, Anderson PL, Doblecki-Lewis S, Bacon O, Chege W, Postle BS, Matheson T, Amico KR, Liegler T, Rawlings MK, Trainor N, Blue RW, Estrada Y, Coleman ME, Cardenas G, Feaster DJ, Grant R, Philip SS, Elion R, Buchbinder S, Kolber MA (2016). Preexposure prophylaxis for HIV infection integrated with municipal- and community-based sexual health services. JAMA Intern Med.

[10] Krakower D, Ware N, Mitty JA, Maloney K, Mayer KH (2014). HIV providers' perceived barriers and facilitators to implementing pre-exposure prophylaxis in care settings: a qualitative study. AIDS Behav.

[11] Krakower DS, Mayer KH (2015). Pre-exposure prophylaxis to prevent HIV infection: current status, future opportunities and challenges. Drugs.

[12] Karris MY, Beekmann SE, Mehta SR, Anderson CM, Polgreen PM (2014). Are we prepped for preexposure prophylaxis (PrEP)? provider opinions on the real-world use of PrEP in the United States and Canada. Clin Infect Dis.

[13] Mera R, McCallister S, Palmer B, Mayer G, Magnuson D, Rawlings K (2016). Truvada (TVD) for HIV pre-exposure prophylaxis (PrEP) utilization in the United States (2013-2015).

[14] Smith D, Van HM, Wolitski R, Stryker J, Hall H, Prejean J, Koenig L, Valleroy L (2015). CDC.

[15] Blumenthal J, Jain S, Krakower D, Sun X, Young J, Mayer K, Haubrich R, CCTG 598 Team (2015). Knowledge is power! increased provider knowledge scores regarding pre-exposure prophylaxis (PrEP) are associated with higher rates of PrEP prescription and future intent to prescribe PrEP. AIDS Behav.

[16] Krakower DS, Mayer KH (2016). The role of healthcare providers in the roll out of preexposure prophylaxis. Curr Opin HIV AIDS.

[17] Seidman D, Carlson K, Weber S, Witt J, Kelly PJ (2016). United States family planning providers' knowledge of and attitudes towards preexposure prophylaxis for HIV prevention: a national survey. Contraception.

[18] Scholl E (2016). Improving outpatient implementation of preexposure prophylaxis in men who have sex with men. J Am Assoc Nurse Pract.

[19] Mimiaga MJ, White JM, Krakower DS, Biello KB, Mayer KH (2014). Suboptimal awareness and comprehension of published preexposure prophylaxis efficacy results among physicians in Massachusetts. AIDS Care.

[20] White JM, Mimiaga MJ, Krakower DS, Mayer KH (2012). Evolution of Massachusetts physician attitudes, knowledge, and experience regarding the use of antiretrovirals for HIV prevention. AIDS Patient Care STDS.

[21] Punyacharoensin N, Edmunds WJ, De Angelis D, Delpech V, Hart G, Elford J, Brown A, Gill ON, White RG (2016). Effect of pre-exposure prophylaxis and combination HIV prevention for men who have sex with men in the UK: a mathematical modelling study. The Lancet HIV.

[22] Zielinski M, Leung S, Akkaya-Hocagil T, Rowe K, Ortega-Peluso C, Smith L (2015). Correlates of routine HIV testing practices: a survey of New York State primary care physicians, 2011. J Acquir Immune Defic Syndr.

[23] Myers JJ, Koester KA, Dufour MK (2011). Barriers and facilitators to enhancing HIV testing in publicly funded primary care clinics: findings from San Francisco. AIDS Educ Prev.

[24] Sharma M, Wilton J, Senn H, Fowler S, Tan DHS (2014). Preparing for PrEP: perceptions and readiness of canadian physicians for the implementation of HIV pre-exposure prophylaxis. PLoS One.

[25] Tripathi A, Ogbuanu C, Monger M, Gibson JJ, Duffus WA (2012). Preexposure prophylaxis for HIV infection: healthcare providers' knowledge, perception, and willingness to adopt future implementation in the southern US. South Med J.

[26] Tellalian D, Maznavi K, Bredeek UF, Hardy WD (2013). Pre-exposure prophylaxis (PrEP) for HIV infection: results of a survey of HIV healthcare providers evaluating their knowledge, attitudes, and prescribing practices. AIDS Patient Care STDS.

[27] Senn H, Wilton J, Sharma M, Fowler S, Tan DH (2013). Knowledge of and opinions on HIV preexposure prophylaxis among front-line service providers at Canadian AIDS service organizations. AIDS Res Hum Retroviruses.

[28] Shaeer K, Sherman E, Shafiq S, Hardigan Patrick (2014). Exploratory survey of Florida pharmacists' experience, knowledge, and perception of HIV pre-exposure prophylaxis. J Am Pharm Assoc (2003).

[29] Smith DK, Koenig LJ, Martin M, Mansergh G, Heneine W, Ethridge S, Morgan M, Mermin J, Fenton F (2014). US Public Health Service.

[30] Centers for Disease ControlPrevention (CDC) (2011). Interim guidance: preexposure prophylaxis for the prevention of HIV infection in men who have sex with men. MMWR Morb Mortal Wkly Rep.

[31] Smith D, Thigpen M, Nesheim S, Lampe M, Paxton L, Samandari T (2012). CDC.

[32] Smith D, Martin M, Lansky A, Mermin J, Choopanya K (2013). CDC.

[33] Kirk MD, Pires SM, Black RE, Caipo M, Crump JA, Devleesschauwer B, Döpfer D, Fazil A, Fischer-Walker CL, Hald T, Hall AJ, Keddy KH, Lake RJ, Lanata CF, Torgerson PR, Havelaar AH, Angulo FJ (2015). Correction: world health organization estimates of the global and regional disease burden of 22 foodborne bacterial, protozoal, and viral diseases, 2010: a data synthesis. PLoS Med.

[34] Liu A, Cohen S, Follansbee S, Cohan D, Weber S, Sachdev D, Buchbinder S (2014). Early experiences implementing pre-exposure prophylaxis (PrEP) for HIV prevention in San Francisco. PLoS Med.

[35] Kelley CF, Kahle E, Siegler A, Sanchez T, Del RC, Sullivan PS, Rosenberg ES (2015). Applying a PrEP continuum of care for men who have sex with men in Atlanta, Georgia. Clin Infect Dis.

[36] (2014). US Public Health Service.

[37] Smith DK, Pan Y, Rose CE, Pals SL, Mehta SH, Kirk GD, Herbst JH (2015). A brief screening tool to assess the risk of contracting HIV infection among active injection drug users. J Addict Med.

[38] Brooke J Usability.

[39] Miles M (1994). Qualitative data analysisa methods sourcebook.

[40] Neuwirth EE, Schmittdiel JA, Tallman K, Bellows J (2007). Understanding panel management: a comparative study of an emerging approach to population care. Perm J.

[41] Chen EH, Bodenheimer T (2011). Improving population health through team-based panel management: comment on “electronic medical record reminders and panel management to improve primary care of elderly patients”. Arch Intern Med.

[42] Chuang E, Ganti V, Alvi A, Yandrapu H, Dalal M (2014). Implementing panel management for hypertension in a low-income, urban, primary care setting. J Prim Care Community Health.

[43] Loo TS, Davis RB, Lipsitz LA, Irish J, Bates CK, Agarwal K, Markson L, Hamel MB (2011). Electronic medical record reminders and panel management to improve primary care of elderly patients. Arch Intern Med.

[44] Kaminetzky CP, Nelson KM (2015). In the office and in-between: the role of panel management in primary care. J Gen Intern Med.

[45] Zhou YY, Unitan R, Wang JJ, Garrido T, Chin HL, Turley MC, Radler L (2011). Improving population care with an integrated electronic panel support tool. Popul Health Manag.

[46] Kirk MD, Pires SM, Black RE, Caipo M, Crump JA, Devleesschauwer B, Döpfer D, Fazil A, Fischer-Walker CL, Hald T, Hall AJ, Keddy KH, Lake RJ, Lanata CF, Torgerson PR, Havelaar AH, Angulo FJ (2015). Correction: world health organization estimates of the global and regional disease burden of 22 foodborne bacterial, protozoal, and viral diseases, 2010: a data synthesis. PLoS Med.

[47] Juusola JL, Brandeau ML, Owens DK, Bendavid E (2012). The cost-effectiveness of preexposure prophylaxis for HIV prevention in the United States in men who have sex with men. Ann Intern Med.

[48] Alistar SS, Owens DK, Brandeau ML (2014). Effectiveness and cost effectiveness of oral pre-exposure prophylaxis in a portfolio of prevention programs for injection drug users in mixed HIV epidemics. PLoS One.

[49] Grant R, Albert L, Hecht J, Buchbinder SP, Weber S, Crouch P, Gibson S, Cohen S, Glidden D (2015). Scale-up of preexposure prophylaxis in San Francisco to impact HIV incidence.

[50] Langhan ML, Riera A, Kurtz JC, Schaeffer P, Asnes AG (2015). Implementation of newly adopted technology in acute care settings: a qualitative analysis of clinical staff. J Med Eng Technol.

[51] Davis MM, Freeman M, Kaye J, Vuckovic N, Buckley DI (2014). A systematic review of clinician and staff views on the acceptability of incorporating remote monitoring technology into primary care. Telemed J E Health.

[52] Main C, Moxham T, Wyatt J C, Kay J, Anderson R, Stein K (2010). Computerised decision support systems in order communication for diagnostic, screening or monitoring test ordering: systematic reviews of the effects and cost-effectiveness of systems. Health Technol Assess.

[53] Nhavoto JA, Grönlund A (2014). Mobile technologies and geographic information systems to improve health care systems: a literature review. JMIR Mhealth Uhealth.

